# Metasurfaces with Freely Varying Height in the Visible Using Grayscale Lithography

**DOI:** 10.1021/acs.nanolett.5c03283

**Published:** 2025-09-11

**Authors:** Daniel N. Shanks, Tobias Wenger, Richard E. Muller, J. Kent Wallace, Daniel W. Wilson

**Affiliations:** Jet Propulsion Laboratory, 53411 California Institute of Technology, Pasadena, California 91109, United States

**Keywords:** metasurfaces, grayscale lithography, diffractive optics, polarization, coronagraphy, exoplanets

## Abstract

Metasurfaces have come to the forefront of optics research due to their unique advantages over conventional glass optics. Metasurfaces are often limited to features that are all the same height due to the use of conventional semiconductor fabrication techniques. In this work, we introduce a grayscale electron-beam lithography step to a well-established atomic layer deposition (ALD)-damascene fabrication process to create dielectric metasurfaces with varying heights that operate in visible wavelengths. We show that this degree of freedom allows for more independent control between the applied dispersion and phase. This comes from quasi-periodic oscillations of both phase and dispersion, which arise as the metasurface top becomes aligned with nodes or antinodes of the standing-wave electric field created at a reflective surface. The enhanced control of these metasurface properties is useful for broadband metasurface applications that require unique tailoring of dispersion, such as retardance correction in large telescopes.

## Graphical abstract

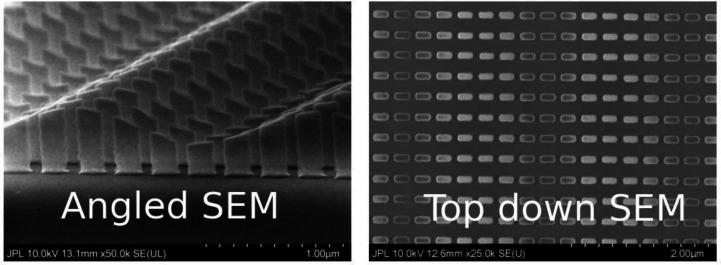

Metasurfaces consist of subwavelength dielectric pillars, or “meta atoms”, which have unique shapes and sizes to control the properties of incident light.^1^ Metasurfaces can perform certain optical transformations of incident light that are either not possible or very challenging by using an equivalent number of conventional glass optics. One of these advantages is precise, nearly arbitrary amplitude and phase profiles with subwavelength precision using the appropriate meta atom placement and design.^2^ Another is control over the polarization properties of light using asymmetrically shaped meta atoms.^3–6^ A third advantage is improved control over the wavelength dependence of the applied phase, or dispersion, to apply an achromatic or a chromatically tailored phase.^7–9^ However, metasurface capabilities can be constrained to a finite subspace within these parameters due to physical limitations of the meta atom shapes and sizes. These restrictions are often imposed by the use of conventional semiconductor nanofabrication processes. One such limitation is often that all meta atoms on an optic must be of the same height. In order to access a wider design space for meta atoms that can enhance the multifunctionality or bandwidth of metasurface capabilities, it is advantageous to find a process that can remove this single-height limitation. There has been some progress in the fabrication of multiheight metasurfaces using two-photon lithography to create custom 3D structures,^10,11^ and promising progress has been made toward the fabrication of complex, inversely designed, 3D photonic nanostructures.^12^ However, this process is limited in feature resolution by the focal spot of the direct writing laser, which limits applications to infrared wavelengths and restricts material choice to photoresists.

In this work, we introduce a grayscale electron-beam (e-beam) lithography step to the established atomic layer deposition (ALD)-damascene metasurface fabrication process,^13^ whereby the surface of the resist profile that defines the metasurface height can be varied in a freeform manner. Grayscale lithography is a well-established process for the fabrication of various diffractive optics with custom 3D surface profiles, such as diffraction gratings,^14–18^ optical vortex masks,^19–21^ or computer generated holograms.^22,23^ The combination of the grayscale lithography process to determine the metasurface height and binary lithography to determine the meta atom lateral dimensions in the same resist is achieved by using two different resist developers with a large gap in dose sensitivity. This two-developer process solves a common problem in grayscale lithography, where the combination of isotropic resist development and the proximity effect from backscattered electrons generally prevents steep vertical features or sharp corners. By using an e-beam process, we achieve meta atom features down to 50 nm, limited by the resolution of the e-beam focal spot (∼10 nm), to create dielectric structures that can operate at visible wavelengths. We then show through both experiment and simulation that varying the meta atom height gains access to a wider parameter space between phase and dispersion, which can be useful for broadband applications.

Polarization aberration correction in large telescopes is an application that can particularly benefit from the advantages gained by freeform height variation of metasurfaces.^24–28^ To correct the retardance over an operational bandwidth of a single coronagraph channel (20% bandwidth), both the retardance and the wavelength dependence of the retardance, which we will refer to as the retardance-dispersion, applied by the metasurface must be tailored to correct the retardance of the as-built telescope. In this work, we show that varying the height of the metasurface allows for more independent control between retardance and retardance-dispersion, enabling more accurate retardance correction over a finite bandwidth.

Figure 1a shows the fabrication process, which consists of an initial grayscale lithography step to define a variable height surface, followed by the ALD-damascene metasurface fabrication for dielectric metasurfaces.^13^ ZEP520A is spun onto either a silicon wafer for test structures or a fused silica substrate with 50 nm of aluminum for optical measurements. We then expose the grayscale pattern in the resist to determine the height profile of the metasurface using electron doses ranging from 5 to 35 μC/cm^2^. Areas that are exposed to lower doses develop at a slower rate, leaving more resist to produce taller meta atoms, and areas at higher dose develop faster to produce shorter ones. Additionally, alignment marks are exposed at high dose to align the following binary lithography step with the initial grayscale lithography step. The sample is developed in the chosen grayscale ZEP520A developer, either pure methyl ethyl ketone (MEK) or 2-butanone or a mixture of MEK/methyl isobutyl ketone^29^ to produce the structure in step 2 of Figure 1a (see Figure S1 for details on the choice of a grayscale developer). The sample is then exposed in the e-beam lithography system a second time with a binary pattern to define the lateral dimensions of the meta atoms using a proximity-effect-corrected dose above 200 μC/cm^2^. The sample is developed in the binary developer, a 1:1 mixture of ZED N50/isopropyl alcohol (IPA).^30^ This weaker developer removes only the area that has been exposed at the higher dose in the second e-beam exposure without significantly altering the grayscale height profile of the remaining resist to create the structure shown in step 3 of Figure 1a.

**Figure 1:**
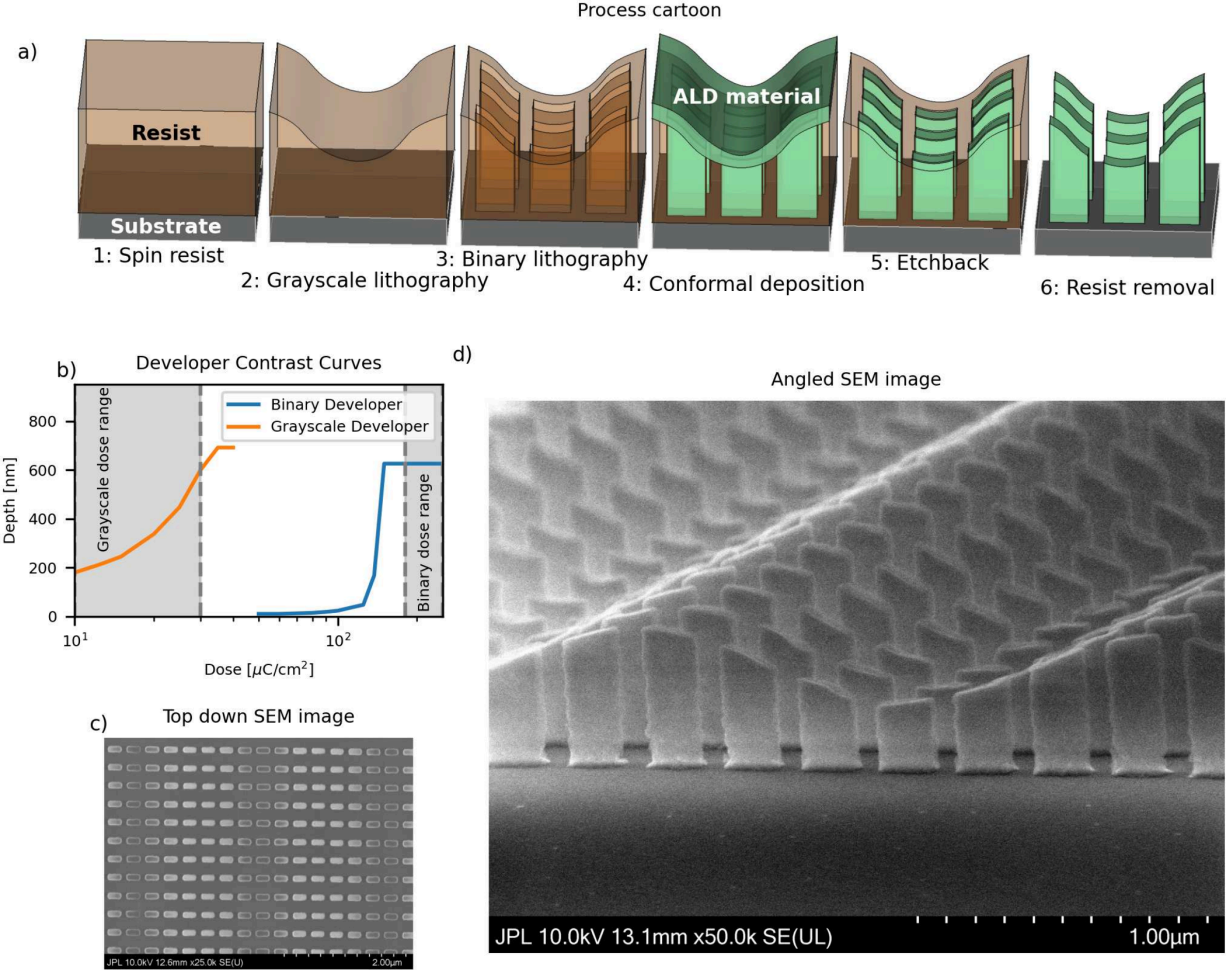
(a) Process cartoon of the multiheight metasurface fabrication process, combining grayscale lithography with a dielectric ALD-damascene process. (b) Contrast curves for ZEP520A development, showing the depth of the removed ZEP520A as a function of the e-beam dose for pure MEK (grayscale developer) and a 1:1 ZED N50/IPA mixture. SEM images of multiheight Al_2_O_3_ metasurfaces, taken from a top-down perspective (c) and at a high angle of incidence (d).

The contrast curves of the two developers are shown in Figure 1b. The large gap in sensitivity between these two developers is the key to obtaining both smoothly varying surface profiles that define the meta atom height in the grayscale step, as well as vertical features that define the meta atom shapes in the binary step. We then conformally deposit a dielectric material, either Al_2_O_3_, as shown in Figure 1, or TiO_2_, as shown in Figure 2, in a thermal ALD process that consists of the metasurface material. The ALD dielectric is overfilled and etched back, and the resist is removed in heated *N*-methyl-2-pyrrolidone to create the final multiheight dielectric structure, shown on the far right of Figure 1a and in SEM images in Figure 1c,d. The multiheight metasurface in Figure 1 is made of Al_2_O_3_ on a silicon wafer, and more nanostructures that show freeform capability in both height and shape can be seen in Figure S2. Such freeform fabrication capability can be highly useful for inversely designed photonic structures.^12,31,32^

**Figure 2:**
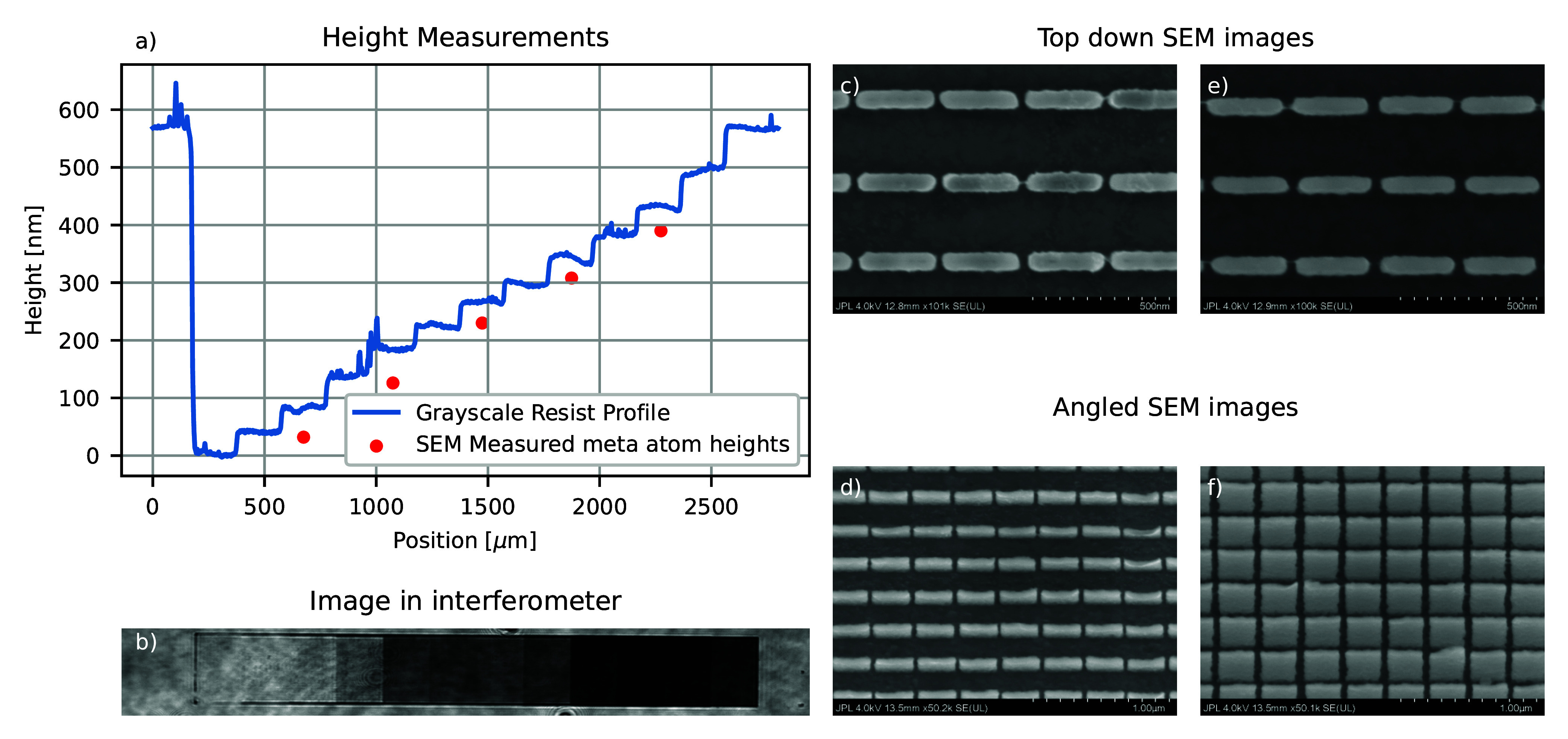
Test device. (a) Height measurements of the metasurface. The blue line shows a profilometer scan of the resist after the grayscale lithography steps, and red dots show the heights of the final meta atoms using angled SEM images. (b) Image of the metasurface in the interferometer, consisting of blocks of identical meta atoms. Shorter meta atoms on the left apply less phase delay, and taller meta atoms on the right apply larger phase delay. (c and d) SEM images of shorter TiO_2_ meta atoms, third column from the left in part b. (e and f) SEM images of taller TiO_2_ meta atoms, ninth column from left in part b.

In order to isolate the effect of the height variation of the metasurface, we fabricated TiO_2_ nanofins and measured the phase and dispersion applied using an interferometer. The lateral dimensions of these nanofins are nearly identical, roughly 275 × 50 nm, and the height of the nanofins varies from 10 to 400 nm tall. Figure 2a shows measurements of the resist height compared to the height of the meta atoms. We measured the grayscale resist height profile using a profilometer after the grayscale lithography step, which gives relative heights of the meta atoms compared to an undeveloped resist. The absolute height of the undeveloped resist was measured by reflectometry. Measurements of the final meta atom heights were taken using SEM images taken at a 30° angle of incidence.

Figure 2b shows an image of the metasurface in the interferometer, where shorter meta atoms are on the left and taller are on the right. Parts c–f of Figure 2 show SEM images of the nanofins at both 0 and 30° angle of incidence, where the height is measured using *ProSEM* image analysis software. The meta atom heights as measured by SEM are shorter than the height of the resist after grayscale lithography by ∼40 nm. This difference could come from many possible steps in the fabrication process. The most likely is that the overgrown dielectric was slightly overetched during the etchback step, step 5 in Figure 1a. Polarization- and wavelength-dependent phase measurements were taken using a custom-built imaging Michelson interferometer, using a white-light laser source with a variable bandpass filter to control the wavelength and linear polarizers on rotation stages at the input and output to control the polarization (see Figures S3 and S4 for further details).

Figure 3 shows the wavelength- and polarization-dependent phase delay applied by the metasurfaces shown in Figure 2, comparing simulation and measurement. Parts a and b of Figure 3 show the simulated phase delay applied by light polarized along the long (a) and short (b) lateral axes of the rectangular meta atoms for meta atoms ranging from 10 to 400 nm tall. Parts c and d of Figure 3 show the measured phase delay of the fabricated metasurface as a function of the polarization and wavelength. Taller meta atoms apply a larger phase shift in both *x* and *y* polarization than shorter ones, and the dispersion of these meta atoms is normal (larger phase applied at shorter wavelengths). Parts e–h of Figure 3 summarize the data from Figure 3a–d, isolating the effect of the height variation. Parts e and f of Figure 3 show the variation of phase applied with changing meta atom height at 600 nm wavelength, while parts g and h of Figure 3 show the simulated and measured dispersion of the meta atoms at 600 nm.

**Figure 3:**
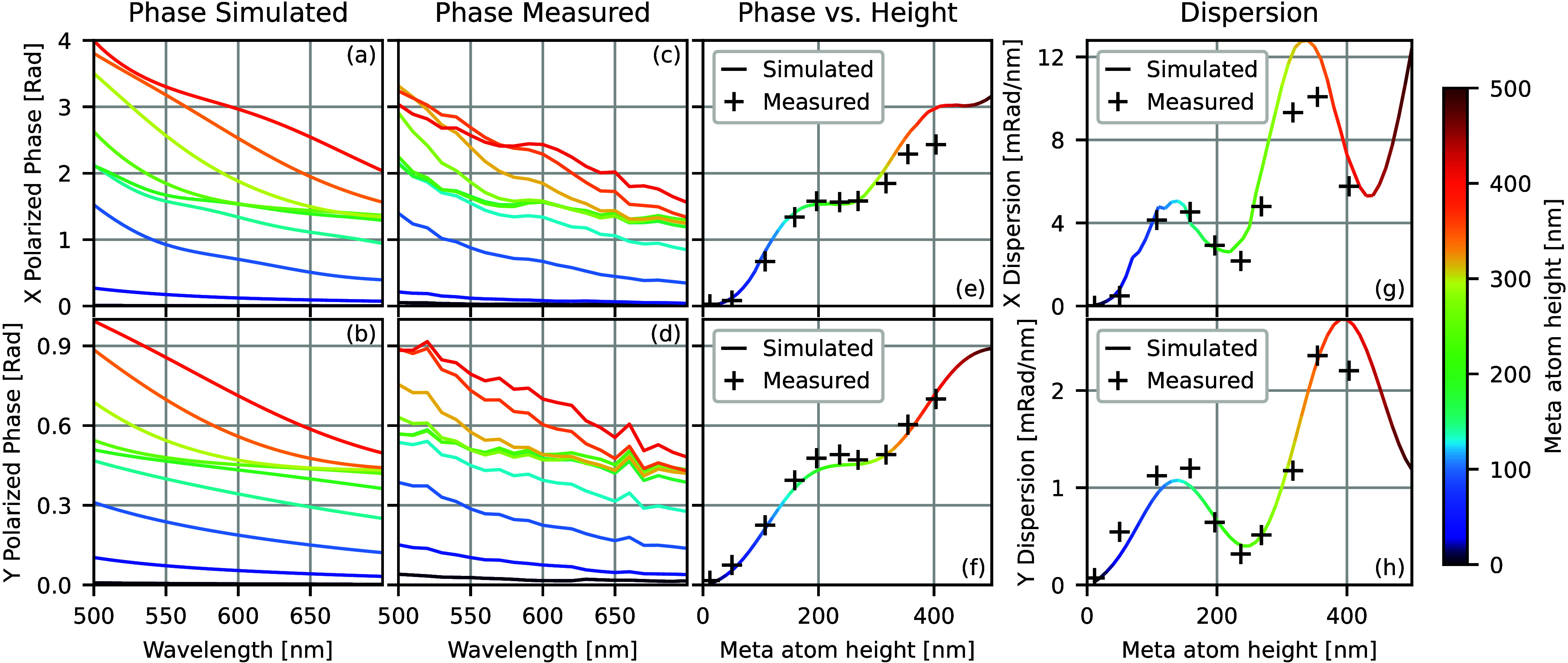
Wavelength- and polarization-dependent phase delay applied by the metasurface as a function of the meta atom height. (a and b) Simulation of the phase applied by meta atoms of varying height using Lumerical FDTD, where each line represents a TiO_2_ meta atom of a different height. (c and d) Measured phase delay applied by fabricated metasurfaces. (e and f) Simulated and measured meta atom phases at 600 nm, representing a vertical line cut of the data in parts a–d. (g and h) Simulated and measured dispersion of meta atoms at 600 nm, calculated as the difference in the phase applied between 560 and 640 nm divided by the bandwidth of 80 nm, approximating the slope of the data in parts a–d.

Both the phase and dispersion show a pseudoperiodic pattern with increasing meta atom height. Parts e and f of Figure 3 show that at roughly half-integer and integer wavelength meta atom heights, the derivative of the phase with respect to height has local minima that are nearly zero. Parts g and h of Figure 3 show that the dispersion features local minima near these same heights. Because the light is reflected off the aluminum surface underneath the metasurface, the electric field intensity forms a standing wave. These local minima occur roughly when the top of the meta atom coincides with a minimum of the electric field intensity of this standing wave (Figure S5). However, these local minima occur at different heights for different polarizations, as can be seen by the dispersion plots being out of phase toward the right-hand side of the plots in Figure 3g,h. Thus, the actual minima occur at not exactly a half- or full-wave physical height, but at a half- or full-wave optical path height. This optical path length is different for different polarizations.

Figure 3 shows that, for reflective metasurfaces, variation of height gives access to a wide range of applied dispersions. Variation of the nanofin shape (width and length) will also provide some capability to control dispersion. To directly explore the dispersion control capability of height variation compared to shape variation, we simulated cuboid nanofins of varying shape and height, both near and far from this half-wave optical height, and plotted their retardance and retardance-dispersion properties in Figure 4.

**Figure 4:**
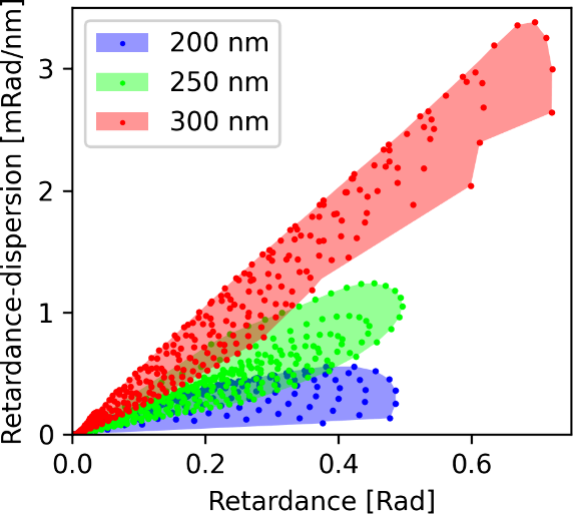
Retardance and retardance-dispersion parameter space accessible to Al_2_O_3_ cuboid nanofins with varying length, width, and height using Lumerical FDTD simulation. The *x* axis shows retardance at the central wavelength (560 nm), and *y* axis plots retardance-dispersion, or the difference in retardance over the 20% operational bandwidth (500–620 nm). Each dot represents the simulation result from a distinct meta atom size, and the shaded region represents the parameter space that can be covered by interpolating within the simulated cuboids for a single height. Each color shows the accessible parameter space for nanofins that are 200 nm tall (blue), 250 nm tall (green), or 300 nm tall (red).

Figure 4 plots the output parameter space between retardance and retardance-dispersion accessible to cuboid nanofin meta atoms of discrete heights. This meta atom library is limited to nanofins with normal retardance-dispersion, where the retardance-dispersion at shorter wavelengths is larger than that of longer wavelengths for all wavelengths in the operational bandwidth (see Figures S6 and S7 for further details). The nanofins at 200 nm height are closest to a half-wave optical height for both polarizations and thus have the lowest retardance-dispersion. Meta atoms that are 300 nm tall are slightly farther away from this half-wave height and thus have higher retardance-dispersion. A metasurface with a single height can only cover a limited area of this parameter space, as represented by a single color in Figure 4. If the metasurface height can vary, it can cover the area of all colors in Figure 4, enclosing a significantly larger area. For retardance correction in large space telescopes, both the magnitude of the retardance as well as the retardance-dispersion of the metasurface must match the inverse of that applied by the telescope to cancel the aberrations. Covering the maximum area within this output space is ideal to minimize the aberrations over a nonzero bandwidth.

In conclusion, we have demonstrated a novel fabrication process that uses a grayscale lithography step to locally determine the meta atom height and a binary lithography step to determine the lateral meta atom shape. We note that the binary lithography step is vertically uniform, which does not enable vertical gaps in individual meta atoms. Additionally, we show that fabricating a meta atom at a half- or full-wave optical height in reflection provides specific advantages. Having a meta atom at this optimal height reduces the dispersion of each individual meta atom, which can be advantageous for a broadband performance of the metasurface if the application aims to apply phase achromatically. Given that the physical height of this integer wave optical height will be different for meta atoms with different lateral dimensions, allowing the metasurface to vary in height across the surface allows all meta atoms remain at this optimal height regardless of shape.

Furthermore, unlocking vertical variation in metasurface fabrication allows for a unique degree of freedom for meta atom design. The additional degree of freedom at the input inherently allows for access to a wider output parameter space of transformations that a metasurface can apply to incident light. The usefulness of this additional freedom will largely depend on the priorities of the application. Given the additional lithographic steps and alignment needed, applications that prioritize performance optimization over mass production are more likely to benefit from this process. One potential advantage from height variation is dispersion control. For the use of a metasurface as a retardance corrector, we show that variation of the height gives access to a larger output space between the retardance and dispersion of retardance. This will improve the performance of a coronagraph by more effectively removing polarization-dependent wavefront errors over the entire operational bandwidth of a coronagraph channel.

## Supplementary Material



## Notes

Methods of simulation, fabrication, and measurement, information on grayscale developers, additional images of height-varying metasurfaces, the interferometer setup used for phase measurements, and further details on the simulation of dispersion of nanofins in Lumerical (PDF)
