# Supplementary material for: Metasurfaces with Freely Varying Height in the Visible Using Grayscale Lithography
Source: Nano Lett. 2025 Sep 11;25(38):14090–5. doi: 10.1021/acs.nanolett.5c03283 (PMC12465001; doi:10.1021/acs.nanolett.5c03283)
Supplement: Supplementary file 1 [file nl5c03283_si_001.pdf]

Supporting information: Metasurfaces with freely varying height in the visible using grayscale lithography

Daniel N. Shanks,* Tobias Wenger, Richard E. Muller, J. Kent Wallace, and Daniel W. Wilson

Jet Propulsion Laboratory, California Institute of Technology, Pasadena, CA 91109

E-mail: dshanks@jpl.nasa.gov

Methods

Metasurface Simulations

Simulations of meta atom phase delays were performed using commercially available Lumerical finite difference time domain (FDTD) simulations of electromagnetic fields. The simulation consists of incident light on a single meta atom with periodic boundary conditions, compared to a simulation of equal volume of air. Mesh is fixed to 5 nm. The index of refraction of the material in the simulation corresponds to the measured index of refraction of the material as deposited on a blank silicon chip in the ALD system. Refractive index measurements are determined using a filmetrics reflectometry system.

Metasurface Fabrication

Substrates consisted of either blank silicon wafers, or 1 inch diameter fused silica uncoated windows from CVI optics. Fused Silica windows were cleaned with diluted micro-90 cleaner and oxygen plasma ashing in a Branson plasma ashing system. Fused silica samples are then coated with 50 nm aluminum in an AJA e-beam evaporator. ZEP520A was spun at 1500 rpm to make a thin film roughly 700 nm thick. For the silicon wafer samples, the ZEP520A is baked at 180°C for 3 min on a hot plate. For the fused silica samples, the ZEP520A is oven baked at 120°C for 30 minutes. A conductive discharge layer (espacer) is then spin-coated on the sample to prevent charging of the resist during exposure, and the resist is exposed to the grayscale pattern at 48 kV in a JEOL 9500 e-beam lithography system. The e-spacer is washed off in DI water, and the sample is developed in the chosen grayscale developer, either MEK:MIBK 2:3 for 30s, or MEK for 10s, and dried with compressed dry air. The sample is then re-coated with espacer, and exposed to the binary pattern in the same JEOL e-beam system. The espacer is washed off in DI water again, and the sample is developer in the binary developer, ZED N50:IPA 1:1 for 60s with sonication, rinsed in IPA and blown dry. The sample is then placed in a BENEQ atomic layer deposition system at 90°C. $TiO_2$2 growth consists of alternating pulses of $TiCl_4$ and water vapor, while Al_2O_3 growth consists of alternating pulses of trimethyl aluminum (TMA) and water vapor. The excess dielectric is etched back using a Unaxis ICP-RIE system with a combination of BCl_3, Cl_2 and Ar gas, in many short cycles to prevent heating of the sample. The resist is removed in NMP heated to 70°C for 2-6 days.

Interferometric phase measurements

The interferometer used for phase measurements of the metasurface consists of a Michelson interferometer. The metasurface sample is on a static stage in one arm of the interferometer. The second arm consists of an aluminum coated mirror on a Physik Instrumente linear piezo stage. The angle of incidence on the beam splitter is kept to ¡10 degrees to avoid excess

polarization effects. Input and output linear polarizers (thorlabs) are in Zaber automated rotation stages. The light from the recombined arms is then re-imaged onto a thorlabs Kiralux camera. The piezo stage is swept through many positions, and an image is captured at each position. The intensity data at each pixel across all images is fourier transformed as a function of piezo stage to obtain the interferometric phase at each pixel, and the phase delay applied by the metasurface is calculated by comparing the phase on the metasurfaces to that on the surrounding area of bare aluminum. Further details are shown in supplementary figures S3 and S4.

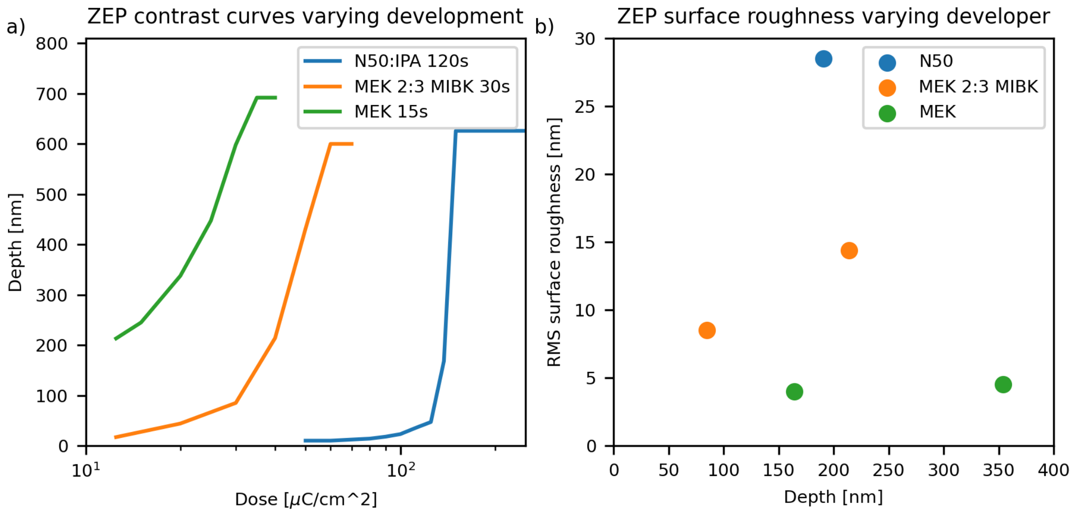

Fig. S1: Contrast curves and surface roughness varying ZEP developer

Figure S1a shows the development rate of ZEP520A in various developers. In this paper, we use MEK and MEK:MIBK 2:3 mixtures as grayscale developers, with comparative advantages and disadvantages. MEK develops the resist significantly faster at the same dose compared to the MEK:MIBK mixture. Thus, achieving an exact depth target when developing by hand can be challenging as the development time must be precise. Additionally, pure MEK will remove unexposed ZEP, reducing the global thickness of the resist. In contrast, the MEK:MIBK mixture develops slower, allowing for more precise development time and more accurate depth. However, this developer shows increased surface roughness of the surface of the grayscale resist profile. Figure S1b shows the surface roughness of the developed ZEP as a function of developer and grayscale development depth. N50 shows the highest surface roughness, followed by the MEK-MIBK mixture, and then pure MEK. Higher surface roughness of the ZEP will translate to more uneven meta atom heights. Thus, for applications that require large variations of surface height, MEK is preferred to maintain a low surface roughness. Alternatively, the MEK:MIBK mixture may be advantageous for applications that require precise meta atom heights with only small variations. Structures in Figs. 1 in the main text and S2 use MEK:MIBK mixture as the grayscale developer, while structures in Fig. 2 in the main text use pure MEK.

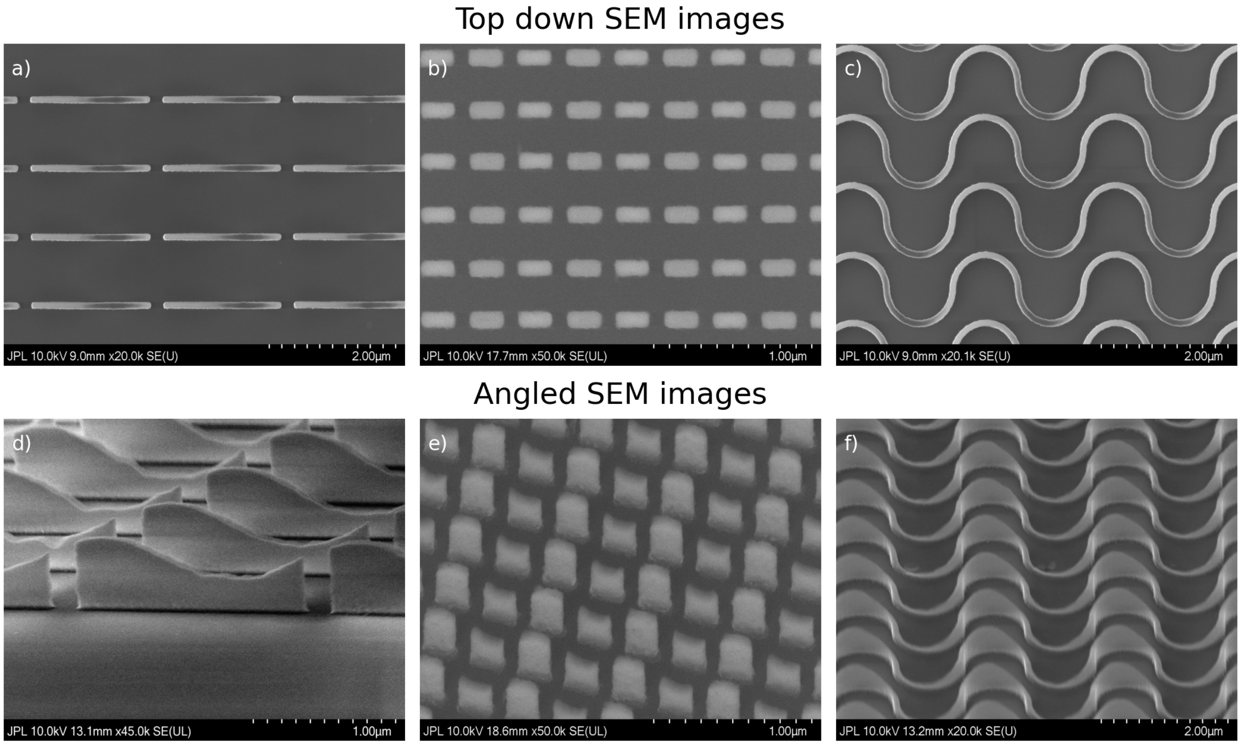

Fig. S2: SEM images of metasurface-like fabricated nanostructures frop a top down perspective (a-c) and angled perspective (d-f), showing freeform fabrication capability in both vertical and lateral dimensions.

Figure S2 shows SEM images of more fabricated nanostructures, showing freeform capability of fabrication.

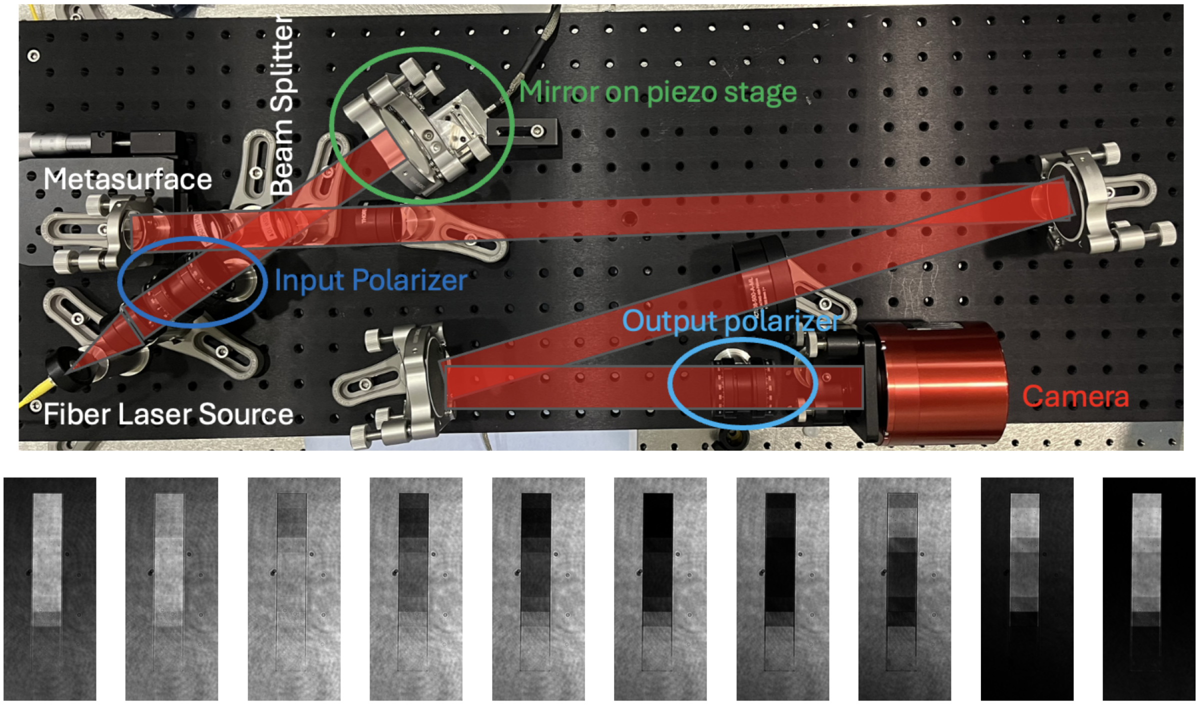

Fig. S3: Top) Michelson interferometer layout. Bottom) Images on the camera with varying piezo stage position, showing areas of constructive and destructive interference.

Figure S3 shows the layout and sample images from the Michelson interferometer used to measure the metasurfaces. Top picture shows an image of the interferometer, with the optical path outlined in red. Light is fed from a SuperK continuum white light laser connected to a fiber source in the set up. The laser uses a SuperK Varia variable bandpass filter to select wavelength. The light is collimated from the fiber, and sent through a polarizer on a rotation stage. The light then hits a beam splitter in the center of the Michelson interferometer, and sent to two arms. The reflected arm goes through a compensator plate to account for the thickness of the beam splitter, and reflects off the metasurface under test back to the beam splitter. The transmitted arm goes through the beamsplitter, and reflects off of a mirror on piezo-electric motion stage. The thickness and material of the beam splitter and compensator plate are matched, such that the optical path length in both arms of the interferometer is equal. This is to ensure that the interference signal over a fintie bandwidth is maximized for all wavebands. The arms are recombined at the beam splitter

and sent through imaging lenses to an output polarizer followed by the camera. All lenses in the system are achromatic doublets, designed for a 400-700 nm range. In order to obtain polarization dependent measurements, both the input and output polarizer are rotated. In order to obtain wavelength dependent measurements, the source laser's bandpass is changed.

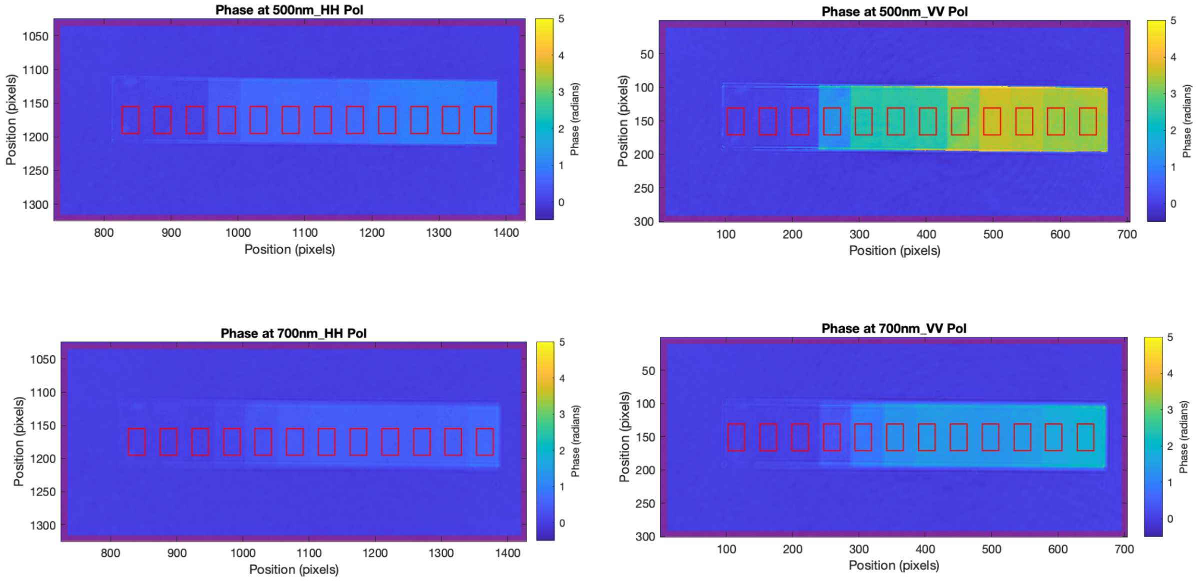

Fig. S4: Phase measurements from the interferometer shown in Figure S3. Top figures show measurement at 500 nm input wavelength, bottom show 700 nm. Left figures show measurement with H polarized light (both input and output polarizers set to H), right figures show V polarized.

Figure S4 shows example phase measurements at different wavelengths and polarizations. Red boxes outline the sub-areas from each different height of metasurfaces that are averaged over to obtain the phase values in Fig. 3 of the main text. Phase measurements are taken relative to the surrounding blank aluminum area where there is no metasurface. The purple outline at the edge of each phase image shows the plane that is flattened to obtain phase measurements relative to the substrate.

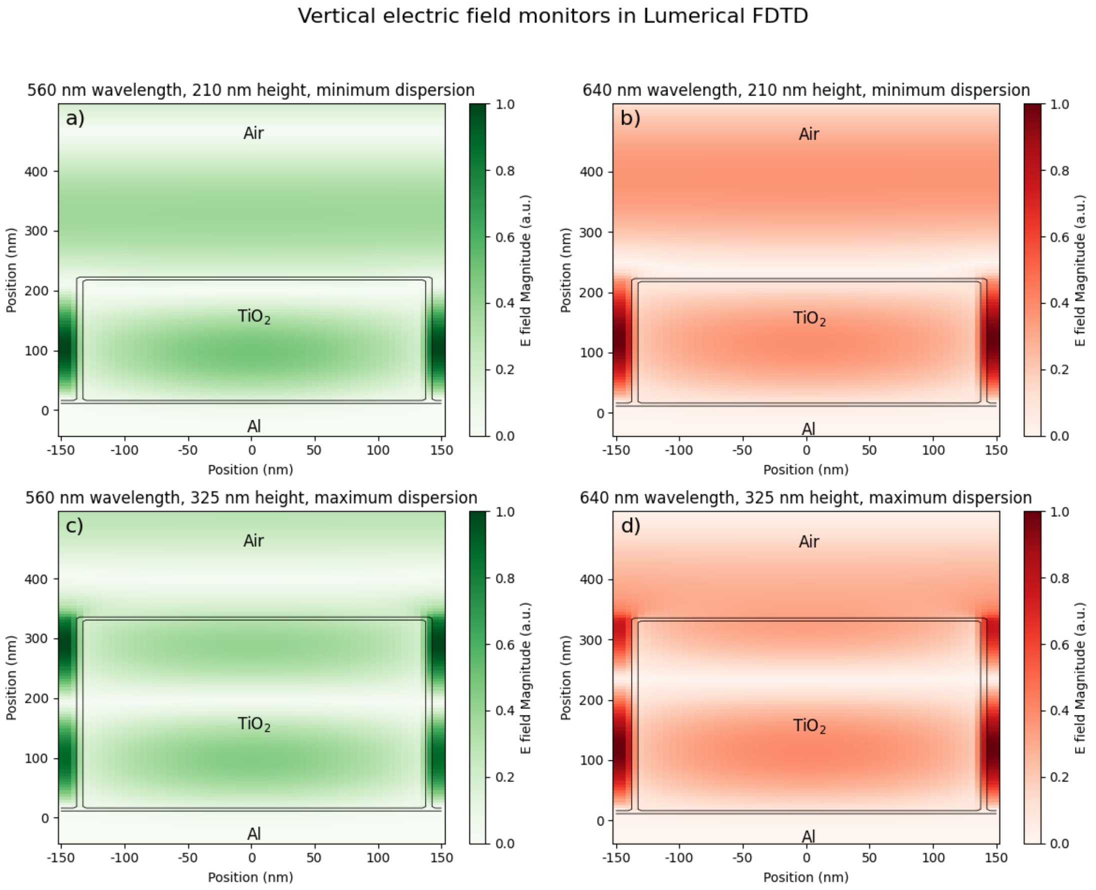

Fig. S5: Vertical electric field profiles in Lumerical FDTD simulation for TiO_2 meta atoms corresponding to the meta atom configuration in the simulations included in Fig. 3 of the main text (275 x 50 nm lateral dimensions, 300 nm period, periodic boundary conditions). Material boundaries are shown by the black lines overlaid on the electric field strength. (a,b) show the electric field intensity for a meta atom near a half wave height, 210 nm, (c,d) show the same for a meta atom of 325 nm height. (a,c) show the simulation using 560 nm light, (b,d) show the same for 640 nm. Simulations use x polarized light, along the long dimension of the meta atom."

Supplementary Figure S5 shows vertical cuts of the normalized electric field magnitude in the Lumerical FDTD simulation near and away from a half wave tall TiO_2 meta atom. Light is incident from the top of the simulation, passes through the TiO_2 meta atom material, and reflects off of the aluminum at the bottom of the simulation, creating a standing wave. Plots a,b, show the data for a meta atom near a half wave height, which correlates to the local minimum dispersion point in Fig. 3(g) of the main text. Plots c,d show the same simulation

for a meta atom of 325 nm height, corresponding to the local maximum dispersion point in Fig. 3(g) of the main text. The color plot of the 210 nm meta atom with minimal dispersion shows that the top of the meta atom is located near an electric field minimum of the standing wave, and in (c,d) the top of the meta atom with maximal dispersion is near an electric field maximum. This highlights the physical effect that leads to the beneficial chromatic tuning capability that can be achieved using multi-height metasurfaces.

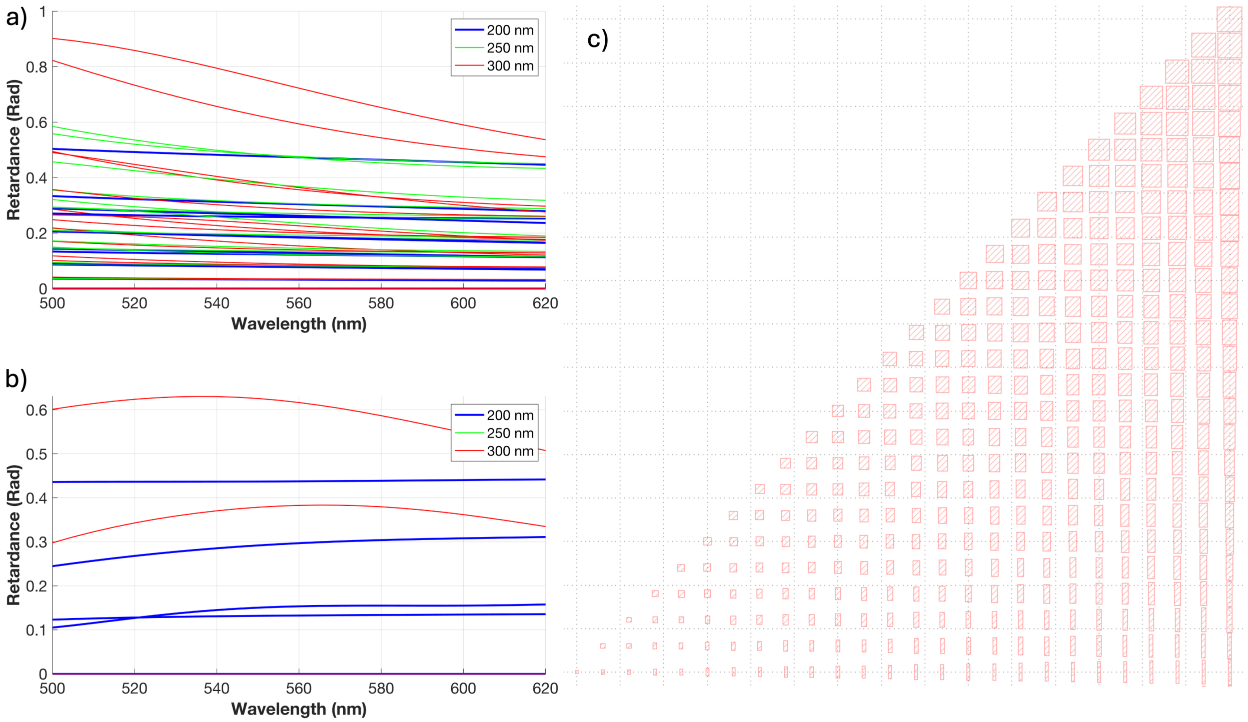

Fig. S6: Retardance as a function of wavelength for a few of the meta atoms included in the library shown in figure 4 (a) and rejected from the library (b) in the main text. c) All meta atom shapes included in the simulation for figure 4 of the main text and S7 of the supporting information.

Figure S6 shows results from the Lumerical simulations that show the retardance and retardance-dispersion of meta atoms in figure 4 of the main text. Simulated meta atom libraries consist of cuboids of Al_2O_3, with a period of 300 nm, all combinations of length and width varying from 35 to 285 nm in 10 nm intervals, as shown in Fig. S6c, and a height of 200, 250 or 300 nm. This data directly shows that meta atoms with 200 nm height (blue) have less dispersion over the operational bandwidth, while meta atoms at 300 nm (red) have larger dispersion. Meta atom libraries are limited to meta atoms with normal dispersion, where retardance at shorter wavelengths is larger than retardance at longer wavelengths for all wavelengths in the operating regime. Top plot shows sample meta atoms included in this library where the retardance-dispersion is normal across the operational bandwidth, bottom plot shows sample meta atoms removed from the library where the retardance-dispersion is anomalous for some or all of the bandwidth. Dispersion of retardance of most

mirror coatings being considered for HWO are largely normal,[1] and thus meta atoms with anomalous dispersion will not be useful for this application. Retardance in early designs for the Habitable Worlds Observatory is less than a radian, and thus full 2-pi phase coverage is not necessary. Lumerical simulations consist a single nanofin of either TiO_2 (Fig. 3 in the main text) or Al_2O_3 for (Fig 4 in the main text, S6, S7) with periodic boundary conditions.

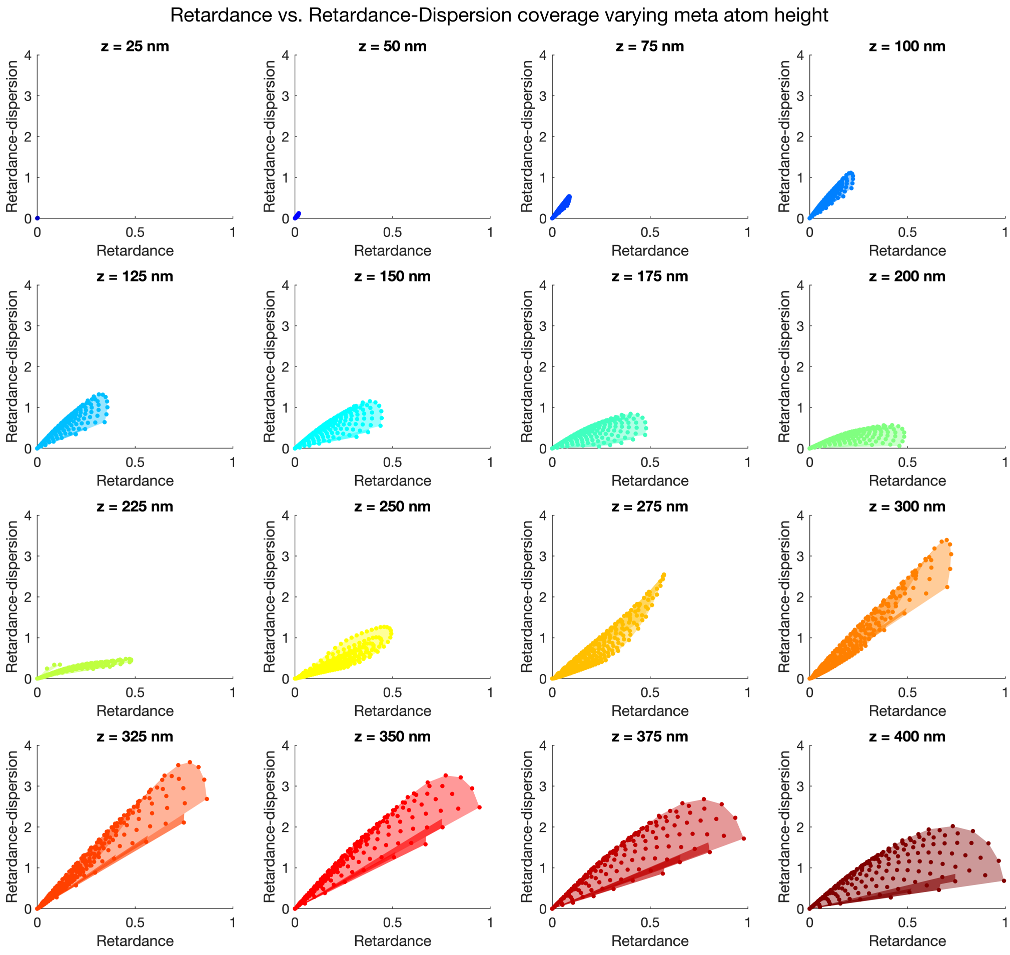

Fig. S7: Full data set of retardance vs. retardance-dispersion phase space coverage for meta atoms with z varying from 25 to 400 nm.

Figure S7 shows the complete data set of retardance vs. retardance-dispersion phase space coverage for for meta atoms from 25 to 400 nm tall in 25 nm steps, corresponding to meta atom shapes shown in figure S6(c).

References

(1) Tuttle, S. et al. Ultraviolet Technology To Prepare For The Habitable Worlds Observatory. 2024; `http://arxiv.org/abs/2408.07242`, arXiv:2408.07242 [astro-ph].
